# The Effects of Oil Content on the Structural and Textural Properties of Cottonseed Butter/Spread Products

**DOI:** 10.3390/foods12224158

**Published:** 2023-11-17

**Authors:** Zhongqi He, Stephen I. Rogers, Sunghyun Nam, K. Thomas Klasson

**Affiliations:** USDA-ARS, Southern Regional Research Center, 1100 Allen Toussaint Blvd., New Orleans, LA 70124, USA; stephen.rogers2@usda.gov (S.I.R.); sunghyun.nam@usda.gov (S.N.); thomas.klasson@usda.gov (K.T.K.)

**Keywords:** butter, cottonseed, glandless, oxidation stability, particle size distribution, texture

## Abstract

Plant-based butters from nuts and seeds have steadily increased in consumer popularity due to their unique flavors and healthy nutritional properties. Oil content is a critical parameter to measure the proper consistency and stability of plant butter and spread products. Previous work has shown that glandless cottonseed can be used to formulate cottonseed butter products to increase the values of cottonseed. As part of the efforts made in the valorization of cottonseed, this work evaluated the effects of oil content on the microstructural and textural properties of cottonseed butter/spread products. While the oil content in the raw cottonseed kernels was 35% of the kernel biomass, additional cottonseed oil was added to make cottonseed butter products with six oil content levels (i.e., 36, 43, 47, 50, 53, and 57%). The values of three textural parameters, firmness, spreadability, and adhesiveness, decreased rapidly in an exponential mode with the increasing oil content. The particle size population in these butter samples was characterized by similar trimodal distribution, with the majority in the middle mode region with particle sizes around 4.5–10 μm. Higher oil content decreased the butter particle size slightly but increased oil separation during storage. The oxidation stability with a rapid oxygen measurement was gradually reduced from 250 min with 36% oil to 65 min with 57% oil. The results of this work provide information for the further optimization of formulation parameters of cottonseed butter products.

## 1. Introduction

Like other plant seeds and tree nuts, cottonseed is rich in human nutrients [1,2,3]. While a whole cottonseed contains about 27% hulls, 8% linters, 4% waste, 45% meals, and 16% crude oil, its dehulled meat (i.e., kernel) contains about 37% crude oil, 41% protein, 10% acid detergent fiber, as well as other carbohydrate and mineral nutrients [4]. In addition, bioactive compounds and peptides with some health benefits have also been found in cottonseed [5,6,7,8,9,10]. On the other hand, the allergic degrees of cottonseed proteins are lower than peanut and tree nuts [11,12,13]. However, traditional cottonseed and its meal products are not suitable for human consumption due to the presence of a toxic polyphenolic chemical (gossypol) in its pigment glands, meaning that this type of cottonseed is also named glanded cottonseed [3,14], unless the gossypol content is lowered [15].

To promote the application of cottonseed products in the food industry, complicated processes are required to lower the gossypol content of the glanded (Gd) cottonseed products below 450 ppm as required by the US Food and Drug Administration’s food standard, or 600 ppm of the allowable limit as per the United Nations Food and Agriculture Organization and the World Health Organization [16,17,18]. Alternatively, efforts have been made toward developing glandless (Gl) cotton varieties with a very low gossypol content [19,20,21]. With such low gossypol contents, the application of cottonseed products in the food industry is very promising. For example, soaked cottonseed kernels have been used to make the traditional Indian drink, “Paruthi Paal”, and novel “cottonseed milk” [22,23]. Recently, response surface methodology has been applied to optimize the extraction conditions for obtaining a high cottonseed milk yield [24]. Cottonseed meal has been co-extruded with corn flour to make nutritional snacks [25,26]. In our research efforts, we have evaluated the impact of the roasting temperature (from 110 to 150 °C) on the chemical profiles of extractable components of cottonseed kernels [27]. Based on the results, we have completed the initial formulation of peanut butter-like products from Gl cottonseed kernels and evaluated their oxidative stability under accelerated storage conditions [28,29].

Indeed, nut and seed butters/spreads have steadily increased in consumer popularity for incorporation into healthy diets and lifestyles in the last few decades. This is because these plant-based butter/spread products are very good sources of protein, fiber, essential fatty acids, and other nutrients with no cholesterol as dairy butter products do [30]. These plant-based butters or spreads are typically made by roasting the nut/seed kernels and then grinding (homogenizing) them with selected additives (e.g., sugar, salt, flavors, and/or emulsifiers). These plant-based butter/spread products have been reported from at least 14 plant sources (i.e., almond, canarium, cashew, cottonseed, flaxseed, hazelnut, macadamia, melon seed, peanut, pistachio, pumpkin seed, sesame, soybean, and sunflower seed) [28,30,31,32,33,34,35]. Recently, olive residue from the typical olive oil extraction process has been tested as a new olive-based spread product enriched with proteins, antioxidant compounds, and functional properties [36]. With strict definitions, the terms “butter” and “spread” have slightly different meanings in terms of the percentage of the main ingredients. The United States Department of Agriculture (USDA) has specified that nut butter comprises at least 90% nuts, while nut spread includes a minimum of 40% nuts dispersed in the continuous oil phase of nut oil [37]. The USDA mandate could also be extended to cover similar plant-seed-based products. That is, plant-based (including both nut/seed) butter refers to a product that contains at least 90% nut/seed ingredients, whereas spread refers to a spreadable product having at least 40% nut ingredients which can be added in various forms, e.g., as nuts, as a paste, and/or as a slurry [30]. For some convenience, a loosely defined alternative term, butter-like spreads, has also been adapted to cover these plant-based butter/spread products without distinction [38]. Hereafter in this work, we will use the general term “cottonseed butter(s)” to refer the peanut butter/spread-like product(s) we made, unless, in some circumstances, the distinct terms of butter and spread are needed for more meaningful analysis and discussion.

Oil is an essential component of butter/spread products, as it is not only a nutrient ingredient but also impacts the texture and spreadability of these products. However, overhigh oil content could result in a product that is more runny than spreadable, with a tendency for oil to separate. For example, an effort has been made to reduce the oil content in pecans from the inherent level of about 70% to 55–60% to make a pecan butter product with proper consistency and stability [39]. On the other hand, external oil is needed for those raw materials with less indigenous oil content. For example, pumpkin seed-based products are made from hull-less pumpkin seed press-cakes or flour which are the by-products of the pumpkin seed oil process [40,41]. In this case, hemp oil in the range of 20–40% was added to produce desirable pumpkin seed-based spreads [40]. Similarly, 16.8% of external cottonseed oil was included in the initial formulation of cottonseed butter-like products due to the low indigenous oil content in Gl cottonseed kernels [28]. However, the influence of the amount of oil added on the product morphology and spreadability has not been fully evaluated yet. Thus, in this work, cottonseed butter/spread products were formulated with six levels of total oil content between 36% and 57%. The specific objectives of this work were (1) to investigate the effect of external cottonseed oil on the microstructure and appearance of the novel cottonseed food products; (2) to compare the shelf stability of these products regarding both rancidity and oil separation, and (3) to determine optimum formulations with respect of the oil content for the manufacture of such spreads.

## 2. Materials and Methods

### 2.1. Materials

Gl cottonseed of NuMex series was provided by Cotton, Inc. (Cary, NC, USA). The kernel products were prepared by mechanical de-hulling and clean-up through a laboratory aspirator in-house [1]. Cottonseed oil (Gefen; Kenover Marketing Corp., Bayonne, NJ, USA), table salt (NaCl; Fisher Scientific, Hampton, NH, USA), and cane sugar (Sucrose; RPI, Mt. Prospect, IL, USA) were acquired from local stores or online.

### 2.2. Cottonseed Butter Preparation

The preparation procedure was modified as per previous reports [28,29]. The butter products were formulated with four ingredients, that is, roasted Gl cottonseed kernels (oil consent 35.0%), external cottonseed oil, table salt and cane sugar. Table 1 lists the specific information of the six butter formulations with the total oil content from 36% to 57%.

To prepare the butter samples, the Gl cottonseed kernels in a metal tray were first roasted in a convection oven (Thermo Scientific Precision Compact Ovens, Waltham, MA, USA) at 140 °C for 30 min. After being cooled down, the roasted kernels were ground with a Waring Commercial Blender (Model WF2211214; Torrington, CT, USA) at a high speed for 3 min, then mixed thoroughly with the required amount of additional cottonseed oil, and fixed amounts of other two ingredients by a spatula. The mixture was blended again with the blender for another 3 min at the high speed, and then transferred to the container of a 200 watts Multi Hand Blender (Braun, Kronberg im Taunus, Germany) for homogenization for 3 min. Those samples were sealed in plastic tubes and kept at 4 °C until analysis.

### 2.3. Color Measurements

The coloric profiles of the six butter products were analyzed with a Spectro2guide spectrophotometer with a built-in calibration standard in the docking station (BYK-Gardner, Columbia, MD, USA) following the same procedure reported before [28]. Parameters determined were the L* value (lightness/darkness), a* value (greenness/redness), and b* value (blueness/yellowness) [32]. The analysis was replicated 3 times.

### 2.4. Textural Parameter Measurement

The butter products were evaluated at 22 °C for firmness, spreadability, and adhesiveness with an EZ-SX texture analyzer (Shimadzu Scientific Instruments, Columbia, MD, USA) [28,39]. A cone-shaped spreading jig set was used to measure the test force required to spread a butter sample between the upper and lower jigs. Test parameters were set as 2 mm s^−1^ for penetration depth of 6 mm and raising speed at 4 mm s^−1^. The test was performed in triplicates.

### 2.5. Particle Size Distribution Measurement

The particle diameter and size distribution of the butter products were evaluated by Partica LA950 laser scattering particle size distribution analyzer (Horiba Scientific, Irvine, CA, USA) [42,43]. A butter sample (0.1 g) was placed into a plastic tube with 5 mL of deionized water, and vortexed for 1 min. A transfer pipette was then used to add the diluted solution dropwise to the distilled water-filled cell of the analyzer. The sample was added until the obscuration was between 0.1 and 0.2. The obscuration referred to the amount of light which was obscured by the sample because of diffraction and absorption. The scattering pattern of the particles was analyzed according to the Mie model, and set refractive indexes for the sample as 1.450 and for water as 1.333 [44,45]. Results are provided as histograms of sizes and cumulative volume percentages.

### 2.6. Scanning Electron Microscopy (SEM)

For the purpose of SEM analysis, the butter products were de-oiled first using hexane extraction and then with overnight drying [28,46]. This process was needed to reduce its possible interference and damage to the microscopy. For SEM analysis, a thin layer of the de-oiled butter particles was gently attached to an 8 mm × 12 mm double-sided sticky carbon tape on an aluminum stud, and a gold coating with a thickness of 5 nm was sputtered onto the sample. The butter sample was then coated with 5 nm thickness of gold. The samples were observed and imaged with a Phenom G6 ProX SEM (Nanoscience Instruments, Phoenix, AZ, USA) at an accelerating voltage of 10 kV.

### 2.7. Butter Storage Stability

The butter storage stability was determined by the oil separation rates over a 60-day storage time [28,47]. These butter samples (5.000 g each) was placed in sealed tubes and kept at room temperature (22 °C). After storage for a given time (i.e., 10, 24, 40 and 60 days), the triplicate tubes of each sample were centrifuged at 1258× *g* for 10 min at 22 °C. The oil component separated by the centrifugation was decanted, and the remaining sold weight was measured and the percentage of oil separation was calculated by the relative change in the sample weight due to the oil decanted.

### 2.8. Oxidation Stability (Rancidity)

The butter samples were analyzed for oxidation stability using RapidOxy 100 m (Anton Paar, Blankenfelde-Mahlow, Germany). A butter sample (1.0 g) was placed in a sample holder and placed in the sealed instrument chamber. The sample was tested with a user-defined program with a start temperature at 25 °C, filling pressure at 700.0 kPa, and target temperature at 120.0 °C (alternatively at 100.0 or 140 °C), and stop criteria at a 10% pressure drop from max pressure at a test temperature of 120 °C and 700 kPa filling pressure until a pressure drop of 10% below maximum pressure was detected. The induction period represented the time in minutes elapsed between the start of the test when oxygen pressure was stabilized at 700 kPa to the pressure drop of 10% from the recorded maximum pressure at the setting temperature per the manufacturer’s operation manual.

### 2.9. Statistical Analysis

All data are presented as mean values of three measurements. Statistical analysis was carried out using Data Analysis Tools embedded in Excel software (version 2310) of Microsoft 365.

## 3. Results and Discussion

### 3.1. Coloic Profile of Cottonseed Butter Products

Table 2 lists the quantitative colorimetric profile of the six butter samples. Data of the a* and b* parameter show the balance between red (positive values) and green (negative values), and yellowness, respectively. The L* value is a parameter to show the comprehensive lightness of a tested sample. In the six samples, the values of a* varied from 4.53 to 6.76. The b* values varied from 24.86 to 30.50, which were much greater than those positive but small a* values. In other words, those butter samples possessed a basic yellow and reddish color tone. The L* values of the six butter products were between 50.87 and 57.27, implicating a moderate brightness of these samples. Oil content seemed to impact the color profile of the six samples. However, no consistent impact patterns were observed between the coloric parameters and the oil content, and the basic yellow hue (b* > a*) did not change in these samples. While color is a visual property of food for consumer preference and purchase decisions, the coloric profiles of the six samples were comparable to those of peanut butter and pumpkin seed spread [28,39,41,48], but the L* values were lower than the value (about 32) of walnut butter in the literature [47].

### 3.2. Textural Characteristics

**Figure 1:**
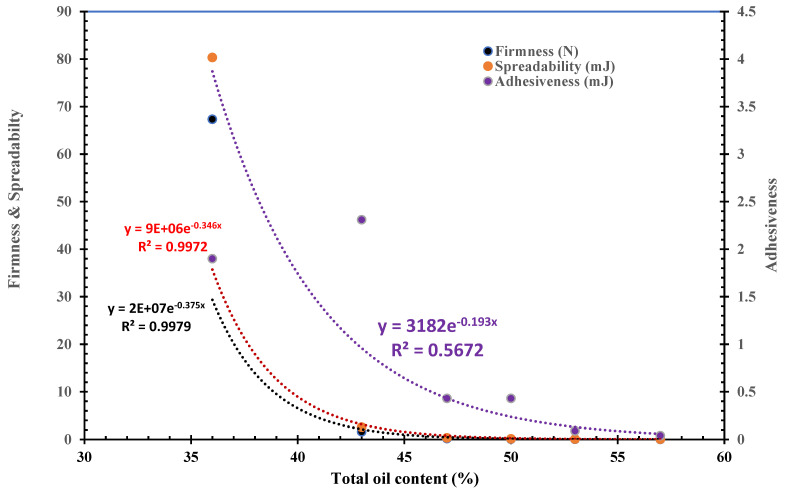
Exponential modelling plots of the three textural parameters of cottonseed butter samples with their total oil contents.

The firmness (peak compression force), spreadability (positive compression work), and adhesiveness (negative work to pull the matching probes apart) of the butter samples were calculated from the force–deformation curve of the texture analysis [28]. The measured values of textural parameters are shown in Table 3. The values of all three parameters dramatically decreased with the increased oil content. As a matter of fact, their changes were so great that the values of the six samples ranged from one or two digits to one or two decimals. In other words, the variances in these data are neither equal, nor normally distributed so that it is impossible to run meaningful statistical significance analysis between these samples without assuming their variances to be the same. Xie et al. [49] observed the fluidity of walnut butter was increased with adding an amount of functional lipids. Their rheology analysis showed that the walnut butter was a non-Newtonian pseudoplastic fluid. Furthermore, Wagener and Kerr [39] reported the exponential decreases in the three parameters with the increased oil content of a pecan butter. Thus, similar exponential modelling was run (Figure 1). The R^2^ values of the exponential decrease in the firmness and spreadability parameters with increased oil contents were quite high (0.9979 and 0.9972, respectively, at significance levels ≤ 0.001). The exponential decrease should be attributed to the fluidity characteristic of the oil component in the butter products. However, the R^2^ value of the exponential plot of the adhesiveness–oil content plot was low (0.4703) which is not statistically significant (*a* > 0.05). This was apparently due to the abnormal value of B36. If this datapoint of B35 was not included, the R^2^ value would have been 0.9720 which was statistically significance at *a* < 0.01. This observation implied that the physicochemical properties involved in the adhesiveness of B36 were not exactly same with other butter samples with higher oil contents. We assumed that while the oil component in high-oil butters (B43 to B57) dominantly contributed the adhesiveness properties, other butter components (e.g., carbohydrate and protein) might have also played certain roles in the adhesiveness with the lower oil content product of B36. Previously, Wagener and Kerr [39] reported that the oil content tested in the range from 50% to 70% decreased values of all the three textural parameters of the pecan butter products with the exponential modes with high R^2^ values (0.927 to 0.988). Therefore, the adhesiveness behavior of our cottonseed butter products seemed to be impacted by oil content not as consistently as that of pecan butter. In contrast, Ahmed and Ali [50] observed that peanut butters with 50% oil content had significantly (*p* ≤ 0.05) lower firmness but higher work of adhesion and stretch (adhesiveness) than peanut butters with 40% oil content The opposite observations in the adhesiveness trends implied both cottonseed and pecan butter products were stiffer and less sticky than the peanut butter and other plant butter products [47,51]. For practical application, B36 and B43 seemed too hard as butter products. The values of B47 fitted for firm butter products. B50 and B53 were good for smooth (creamy) butters. B57 may serve as a more spreadable product.

### 3.3. SEM Analysis of Butter Particle Morphology

**Figure 2:**
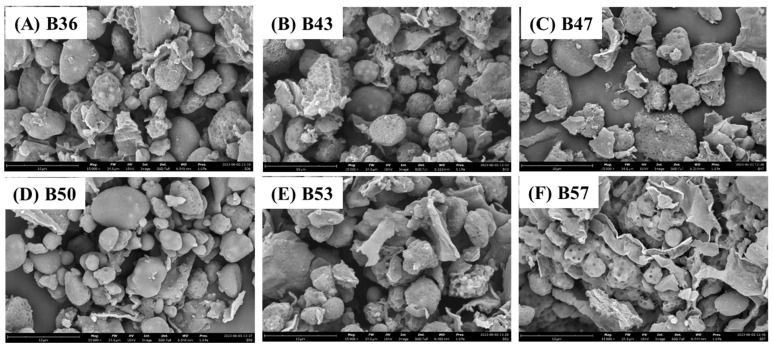
SEM images of the six de-oiled cottonseed butters (15,000× magnification).

After being de-oiled, the images of the butter particle samples were checked by SEM (Figure 2). Generally, most particles in these butter samples are spherical shapes with either smooth or rougher surfaces. Some particles were in the forms of flat and curving sheets, implying their constituents might be non-protein materials of the cottonseed. These observations were similar to the morphology of the particles in cottonseed butter and other cottonseed products in the literature [28,52,53]. On the other hand, the impact of the oil content on the cottonseed butter particles were also observed. Compared to B36, B43 and B47, particles of B50 appeared smoother and smaller, indicating a high degree of homogenization. Those de-oiled particles in B53 and B57 showed the void (pit)-rich spherical surfaces with more coalescent sheets, which may be formed during the de-oil processing with the high oil content in the two butter samples. The difference observations in the surface smoothness of the six de-oiled samples demonstrated the oil component playing a critical role in smoothing and stabilizing of oil seed products [1,51,54].

### 3.4. Particle Size Distribution

**Figure 3:**
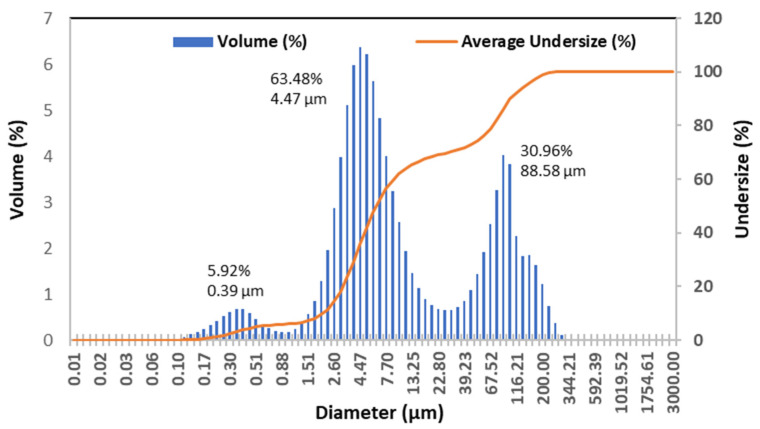
Particle size histogram of B50 with trimodal (three peak) features.

The particle size distribution patterns of the six samples were similar so that a representative distribution histogram of B50 is shown in Figure 3. The histogram reveals that the cottonseed butter particles possess a trimodal distribution pattern with a breadth extent from 0.1 to 350 μm. The three size groups are centered at 0.39 (Peak 1), 4.47 (Peak 2) and 88.58 (Peak 3) μm with relative volumes of 5.92, 63.76, and 30.32% of total particles, respectively. It is typical that plant butter spreads and paste contain the particles of the uneven sizes, and have continuous particle size distributions [45]. The trimodal size distribution patterns are frequently observed in butter and paste products [43,45,46,55]. Tetramodal particle size distributions are also observed in some products with lower grinding times [43,46]. While the relative volumes of the medium particles (Peak 2) were dominant (61–66%) in all samples, those data showed that bigger particles were in low and high oil content samples (Table 4). In other words, change in the oil content seemed not to alter the distribution patterns of the cottonseed butter products, but to change the particle size range and relative volume.

### 3.5. Butter Shelf Stability (Oil Separation during Storage)

**Figure 4:**
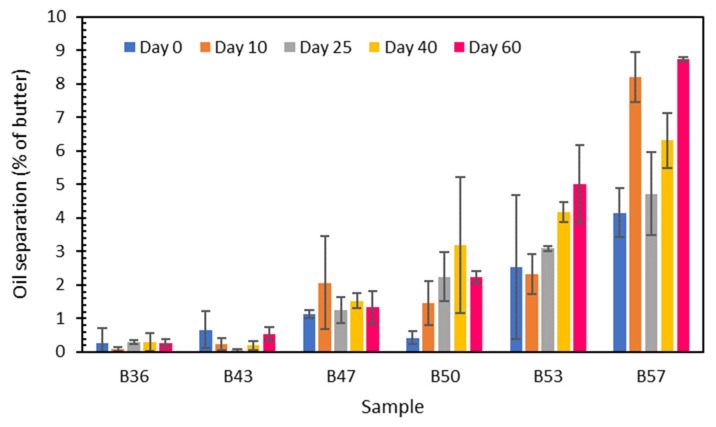
Oil separation rates of six cottonseed butter samples during storage at room temperature (25 °C). Data are presented as averages with standard deviation bars (n = 3).

The shelf stability of the cottonseed butter products was evaluated by oil separation during the storage of the butter products at room temperature over 60 days (Figure 4). The rate of oil separation increased with the increasing oil content. Oil separation also increased with storage time, especially for the high oil content products, B53 and B57. For B36, B43, and B47, the oil that separated from the butter bodies was not more than 2% of the butter weight at any time in the 60-day storage period. The highest oil separation of B50, B53, and B57 during the storage period was 3.2, 5.0, and 8.7% of butter weight, respectively, doubled from their initial oil separation rate without storage. The oil separation rate was comparable to that (2–8%) of sesame pastes reported by Hou et al. [45], but less than that of walnut butter (15–30%) [47], 11–13% separation in peanut butter by Tanti et al. [51], and 11% in sesame paste by Saatchi et al. [56].

### 3.6. Oxidation Stability (Rancidity)

**Figure 5:**
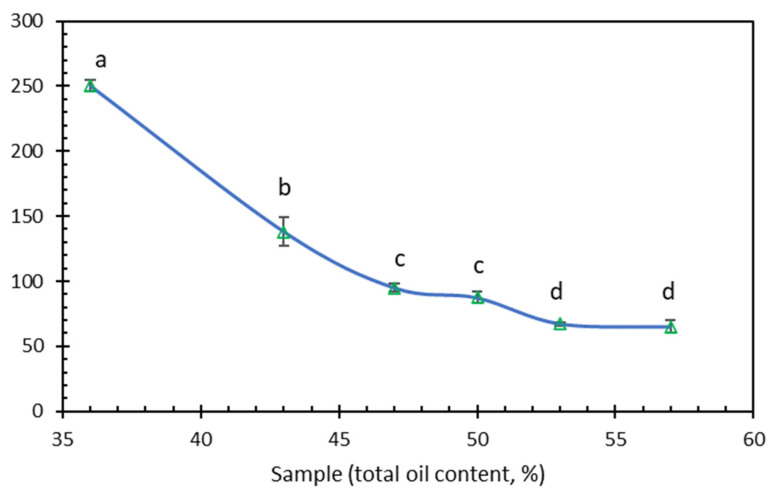
Oxidation stability (oxidation induction time at 120 °C) impacted by the total oil content in the six cottonseed butter products. Data are presented as averages with standard deviation bars (n = 3). Different letters above the data points indicate these values are significantly different statistically (*p* ≤ 0.05).

The oxidation stability was measured by an Anton Paar RapidOxy 100 m. The meter accelerates the oxidation process by using increased temperature and oxygen pressure [57,58]. The induction period, which is the total time between the heating of the test chamber until the 10% pressure drop, can be used as a measure of the oxidative stability of the sample (Figure 5). In the six cottonseed butter products, the oxidation stability steadily was gradually reduced from 250 min with 36% oil to 65 min with 57% oil, indicating the oil ingredient was the key component of rancidity of these butter products. Using the same type of measurements, Jovanović et al. [58] reported oxidation stability of white chocolate products with an oxidation induction time as long as 680 min due to increasing polyphenol contents. To calculate the shelf life per the oxidation stability, we further measured the oxidation induction time of B50 at 100, 120, and 140 °C. A linear equation ln(t[min]) = 8400.00 K/T-17.00 was drawn per the produced data. The oxidation stability of B50 at common temperature (shelf life) was then calculated from the linear equation as 9 days at 40 °C, 34 days and 14 h at 25 °C, and about 56 days at 20 °C. The 34.5-day shelf life of B50 at 25 °C also corresponded to the highest oil separation rate around the 40-day storage time (Figure 4). If needed, synthetic food-grade polymers or natural antioxidant extracts could be applied to increase the oil stabilization and shelf life of the cottonseed butter products, as are used for peanut butter and soybean oil [51,57]. Another option is the replacement of the external cottonseed oil addition by a more stable food oil product [59], such as palm oil [54,60], or mango kernel oil [61].

## 4. Conclusions

Glandless cottonseed kernels with additional cottonseed oil could be used to make plant-butter-like food spread products. As an essential ingredient in the cottonseed butter products, the oil component not only provided nutrients and energy of the products, but also played important roles in the textural properties and butter stability. While oil content did not alter much the appearance (color) of the butter products, high oil content generally improved the textural properties, but lowered the butter stability. On testing the total oil content from 36 to 57%, our data suggested the butter products with 47–50% oil content showed characteristics of firm and smooth butter products with reasonable storage stability. Those products with oil contents ranging between 53 and 57% might serve as creamy spread products with shorter shelf-life times.

## Figures and Tables

**Table 1:** Cottonseed butter formulation.

Butter Name	Cottonseed Kernel (%)	External Cottonseed Oil (%)	Table Salt (%)	Cane Sugar (%)
B36 ^1^	85.8	6.0	0.7	7.5
B43	75.1	16.7	0.7	7.5
B47	68.9	22.9	0.7	7.5
B50	64.3	27.5	0.7	7.5
B53	59.7	32.1	0.7	7.5
B57	53.5	38.3	0.7	7.5

^1^ The numbers in the sample names indicate the total oil content in the samples. The internal oil content in the cottonseed kernels was 35.0%.

**Table 2:** Colorimetric parameters (L*, a*, and b*) of cottonseed butter products.

	L*	a*	b*
B36	54.37 ± 0.02 d ^1^	5.96 ± 0.01 c	25.88 ± 0.01 e
B43	57.09 ± 0.01 b	5.19 ± 0.00 e	26.17 ± 0.00 d
R47	55.22 ± 0.11 c	5.44 ± 0.01 d	24.86 ± 0.12 f
B50	57.27 ± 0.01 a	4.53 ± 0.00 f	28.49 ± 0.02 c
B54	51.21 ± 0.00 e	6.76 ± 0.01 a	30.50 ± 0.01 a
B57	50.87 ± 0.01 f	6.21 ± 0.01 b	29.91 ± 0.02 b

^1^ Different letters in the same column indicate these values statistically significantly different (*p* ≤ 0.05) by Fisher’s LSD Test.

**Table 3:** Textural properties of six cottonseed butter products. Data are present in the format of average ± standard deviation (n = 3).

	Firmness (N)	Spreadability (mJ)	Adhesiveness (mJ)
B36	67.33 ± 18.16	80.32 ± 19.35	1.59 ± 0.05
B43	1.62 ± 0.63	2.58 ± 1.07	1.68 ± 0.06
B47	0.20 ± 0.03	0.34 ± 0.06	0.38 ± 0.06
B50	0.08 ± 0.01	0.13 ± 0.03	0.16 ± 0.04
B53	0.05 ± 0.01	0.09 ± 0.02	0.06 ± 0.03
B57	0.03 ± 0.01	0.07 ± 0.01	0.04 ± 0.01

**Table 4:** Comparison of the particle size ranges and relative volumes of trimodal distribution peaks of the six cottonseed butter products.

	Peak 1			Peak 2			Peak 3		
	Range (μm)	Peak (μm)	Volume (%)	Range (μm)	Peak (μm)	Volume (%)	Range (μm)	Peak (μm)	Volume (%)
B36	<1.00	0.45	8.68 ab ^1^	1.0–44.9	8.82	63.70 b	>44.9	229.1	27.62 b
B43	<1.00	0.39	13.64 a	1.0–34.3	5.12	65.11 ab	>34.3	88.58	21.35 b
B47	<0.88	0.39	6.83 bc	0.88–26.1	4.47	65.92 ab	>26.1	88.58	27.25 b
B50	<0.88	0.39	5.92 bc	0.88–26.1	4.47	63.76 ab	>26.1	88.58	30.32 ab
B53	<0.88	0.39	3.84 bc	0.88–29.1	4.47	68.29 a	>26.1	101.5	27.87 ab
B57	<0.88	0.39	2.47 c	0.88–29.1	4.47	60.54 b	>26.1	101.5	36.99 a

^1^ Different letters in the same volume column indicate these values statistically significantly different (*p* ≤ 0.05) by Fisher’s LSD Test.

## Data Availability

The data used to support the findings of this study can be made available by the corresponding author upon request.

## References

[1] He Z., Nam S., Zhang H., Olanya O.M. (2022). Chemical composition and thermogravimetric behaviors of glanded and glandless cottonseed kernels. Molecules.

[2] He Z., Zhang H., Olk D.C. (2015). Chemical composition of defatted cottonseed and soy meal products. PLoS ONE.

[3] Kumar M., Hasan M., Choyal P., Tomar M., Gupta O.P., Sasi M., Changan S., Lorenzo J.M., Singh S., Sampathrajan V. (2022). Cottonseed feedstock as a source of plant-based protein and bioactive peptides: Evidence based on biofunctionalities and industrial applications. Food Hydrocoll..

[4] NCPA National Cottonseed Products Association-The Products. https://www.cottonseed.com/products/.

[5] He Z., Nam S., Liu S., Zhao Q. (2023). Characterization of the nonpolar and polar extractable components of glanded cottonseed for its valorization. Molecules.

[6] He Z., Zhang D., Mattison C.P. (2022). Quantitative comparison of the storage protein distribution in glandless and glanded cottonseeds. Agric. Environ. Lett..

[7] Cao H., Sethumadhavan K. (2022). Identification of Bcl2 as a Stably Expressed qPCR Reference Gene for Human Colon Cancer Cells Treated with Cottonseed-Derived Gossypol and Bioactive Extracts and Bacteria-Derived Lipopolysaccharides. Molecules.

[8] Song W., Kong X., Hua Y., Li X., Zhang C., Chen Y. (2020). Antioxidant and antibacterial activity and in vitro digestion stability of cottonseed protein hydrolysates. LWT.

[9] Cao H., Sethumadhavan K., Bland J.M. (2018). Isolation of cottonseed extracts that affect human cancer cell growth. Sci. Rep..

[10] Ma M., Ren Y., Xie W., Zhou D., Tang S., Kuang M., Wang Y., Du S.-K. (2018). Physicochemical and functional properties of protein isolate obtained from cottonseed meal. Food Chem..

[11] Mattison C.P., He Z., Zhang D., Dupre R., Lloyd S.W. (2023). Cross-serological reaction of glandless cottonseed proteins to peanut and tree nut allergic IgE. Molecules.

[12] Tan C.F., Kwan S.H., Lee C.S., Soh Y.N.A., Ho Y.S., Bi X. (2022). Cottonseed meal protein isolate as a new source of alternative proteins: A proteomics perspective. Int. J. Mol. Sci..

[13] Atkins F.M., Wilson M., Bock S.A. (1988). Cottonseed hypersensitivity: New concerns over an old problem. J. Allergy Clin. Immunol..

[14] Sihag M.K., Patel A., Kumar V. (2021). Cottonseed (*Gossypium hirsutum*). Oilseeds: Health Attributes and Food Applications.

[15] Kadam D.M., Kasara A., Parab S.S., Mahawar M.K., Kumar M., Arude V. (2023). Optimization of process parameters for degossypolisation of de-oiled cottonseed cake by response surface methodology (RSM). Food Humanit..

[16] Dabbour M., Hamoda A., Mintah B.K., Wahia H., Betchem G., Xu H., He R., Ma H. (2023). Ultrasonic-aided extraction and degossypolization of cottonseed meal protein: Optimization and charascterization of functional traits and molecular structure. Ind. Crops Prod..

[17] Kumar M., Potkule J., Patil S., Saxena S., Patil P.G., Mageshwaran V., Punia S., Varghese E., Mahapatra A., Ashtaputre N. (2021). Extraction of ultra-low gossypol protein from cottonseed: Characterization based on antioxidant activity, structural morphology and functional group analysis. LWT.

[18] Zhang Z., Yang D., Liu L., Chang Z., Peng N. (2022). Effective gossypol removal from cottonseed meal through optimized solid-state fermentation by Bacillus coagulans. Microb. Cell Factories.

[19] Zhang J., Wedegaertner T. (2021). Genetics and breeding for glandless upland cotton with improved yield potential and disease resistance: A review. Front. Plant Sci..

[20] Zhang J., Wedegaertner T., Idowu O.J., Flynn R., Hughs S.E., Jones D.C. (2016). Registration of ‘NuMex COT 15 GLS’glandless cotton. J. Plant Registr..

[21] Gao W., Zhu X., Ding L., Xu B., Gao Y., Cheng Y., Dai F., Liu B., Si Z., Fang L. (2022). Development of the engineered “glanded plant and glandless seed” cotton. Food Chem. Mol. Sci..

[22] Kumar M. (2019). Paruthi Paal, a nutrient-rich healthy drink from cottonseed: An Indian delicacy. J Ethn. Foods.

[23] Thirukkumar S., Hemalatha G., Vellaikumar S., Murugan M., Amutha S. (2021). Influence of enzymes and extraction conditions on high yield of cottonseed milk. J. Environ. Biol..

[24] Subramani T., Ganapathyswamy H., Sampathrajan V., Sundararajan A. (2022). Optimization of extraction parameters to improve cottonseed milk yield and reduce gossypol levels using response surface methodology (RSM). J. Food Process. Preserv..

[25] Reyes-Jáquez D., Casillas F., Flores N., Cooke P., Licon E.D., Soto A.S., González I.A., Carreón F.O.C., Roldán H.M. (2014). Effect of glandless cottonseed meal content on the microstructure of extruded corn-based snacks. Adv. Food Sci..

[26] Reyes-Jáquez D., Casillas F., Flores N., Andrade-González I., Solís-Soto A., Medrano-Roldán H., Carrete F., Delgado E. (2012). The effect of glandless cottonseed meal content and process parameters on the functional properties of snacks during extrusion cooking. Food Nutrit. Sci..

[27] He Z., Liu S., Nam S., Klasson K.T., Cheng H.N. (2023). Molecular level characterization of the effect of roasting on the extractable components of glandless cottonseed by Fourier transform ion cyclotron resonance mass spectrometry. Food Chem..

[28] He Z., Cheng H.N., He J. (2023). Initial formulation of novel peanut butter-like products from glandless cottonseed. Foods.

[29] He Z., Nam S., Klasson K.T. (2023). Oxidative stability of cottonseed butter products under accelerated storage conditions. Molecules.

[30] Gorrepati K., Balasubramanian S., Chandra P. (2015). Plant based butters. J. Food Sci. Technol..

[31] Dimić E.B., Vujasinović V.B., Radočaj O.F., Borić B.D. (2013). Sensory evaluation of commercial fat spread based on oil seeds and walnut. Acta Period. Technol..

[32] Shakerardekani A., Karim R., Ghazali H.M., Chin N.L. (2013). Development of pistachio (*Pistacia vera* L.) spread. J. Food Sci..

[33] Tuhumury H.C.D., Souripet A., Pattiwael K.J. (2023). Production of Canarium (*Canarium indicum* L.) Butter with Different Sugar Concentrations. J. Appl. Agric. Sci. Technol..

[34] Sahin E., Erem E., Güzey M., Kesen M.S., Icyer N.C., Ozmen D., Toker O.S., Cakmak H. (2022). High potential food wastes: Evaluation of melon seeds as spreadable butter. J. Food Process. Preserv..

[35] Ghosal S., Bhattacharyya D.K., Bhowal J. (2022). Production, characterization, and storage stability of nutritionally enriched flaxseed-based spread. J. Food Process. Preserv..

[36] Andreou V., Chanioti S., Stergiou P., Katsaros G. (2021). Valorization of the Olive Oil Production Residue: Healthy Ingredient for Developing High Value-Added Spread. Sustainability.

[37] USDA (2019). USDA Commercial Item Description: Nut Butters and Nut Spreads.

[38] Ningtyas D.W., Prakash S., Bhandari B.R., Gaiani C. (2023). Chapter 8—Plant-based butter like spreads. Engineering Plant-Based Food Systems.

[39] Wagener E.A., Kerr W.L. (2018). Effects of oil content on the sensory, textural, and physical properties of pecan butter (*Carya illinoinensis*). J. Texture Stud..

[40] Radoĉaj O.F., Dimić E.B., Vujasinović V.B. (2011). Optimization of the texture of fat-based spread containing hull-less pumpkin (*Cucurbita pepo* L.) seed press-cake. Acta Period. Technol..

[41] Nikolić I., Dokić L., Rakić D., Tomović V., Maravić N., Vidosavljević S., Šereš Z., Šoronja-Simović D. (2018). The role of two types of continuous phases based on cellulose during textural, color, and sensory characterization of novel food spread with pumpkin seed flour. J. Food Process. Preserv..

[42] Muresan V., Danthine S., Racolta E., Muste S., Blecker C. (2014). The influence of particle size distribution on sunflower tahini rheology and structure. J. Food Process Eng..

[43] Çiftçi D., Kahyaoglu T., Kapucu S., Kaya S. (2008). Colloidal stability and rheological properties of sesame paste. J. Food Eng..

[44] Rozalli N.M., Chin N., Yusof Y. (2015). Grinding characteristics of Asian originated peanuts (*Arachishypogaea* L.) and specific energy consumption during ultra-high speed grinding for natural peanut butter production. J. Food Eng..

[45] Hou L., Li C., Wang X. (2020). The colloidal and oxidative stability of the sesame pastes during storage. J. Oleo Sci..

[46] Norazatul Hanim M., Chin N., Yusof Y. (2016). Effects of grinding time on rheological, textural and physical properties of natural peanut butter stored at different temperatures. J. Texture Stud..

[47] Shahidi-Noghabi M., Naji-Tabasi S., Sarraf M. (2019). Effect of emulsifier on rheological, textural and microstructure properties of walnut butter. J. Food Measur. Character..

[48] Pattee H.E., Giesbrecht F.G., Young C.T. (1991). Comparison of peanut butter color determination by CIELAB L*, a*, b* and Hunter color-difference methods and the relationship of roasted peanut color to roasted peanut flavor response. J. Agric. Food Chem..

[49] Xie Y., Jiang N., Su H., Tan F., Cheng X., Wang J., Hu H. (2023). Effect of functional lipids on the quality of walnut butter prepared from defatted walnut meal by ball mill grinding. Authorea.

[50] Ahmed E., Ali T. (1986). Textural quality of peanut butter as influenced by peanut seed and oil contents. Peanut Sci..

[51] Tanti R., Barbut S., Marangoni A.G. (2016). Oil stabilization of natural peanut butter using food grade polymers. Food Hydrocoll..

[52] He Z., Cheng H.N., Olanya O.M., Uknalis J., Zhang X., Koplitz B.D., He J. (2018). Surface characterization of cottonseed meal products by SEM, SEM-EDS, XRD and XPS analysis. J. Mater. Sci. Res..

[53] Delgado E., Valles-Rosales D., Pámanes-Carrasco G., Cooke P., Flores N. (2021). Structural, rheological and calorimetric properties of an extruded shrimp feed using glandless cottonseed meal as a protein source. J. Aquac. Res. Develop..

[54] Aryana K.J., Resurreccion A.V.A., Chinnan M.S., Beuchat L.R. (2000). Microstructure of peanut butter stabilized with palm oil. J. Food Process. Preserv..

[55] Mureşan V., Danthine S., Bolboacă S.D., Racolţa E., Muste S., Socaciu C., Blecker C. (2015). Roasted sunflower Kernel paste (Tahini) stability: Storage conditions and particle size influence. J. Am. Oil Chem. Soc..

[56] Saatchi A., Kiani H., Labbafi M. (2022). Stabilization activity of a new protein–carbohydrate complex in sesame paste: Rheology, microstructure, and particle size analysis. J. Sci. Food Agric..

[57] Hassan M.A., El-Sayed Hassan Shaltout O.E., Mohamed A. (2022). Improving the thermoxidative stability of soybean oil using some herbal extracts. J. Adv. Agric. Res..

[58] Jovanović P., Pajin B., Lončarić A., Jozinović A., Petrović J., Fišteš A., Zarić D., Tumbas Šaponjac V., Ačkar Đ., Lončarević I. (2022). Whey as a carrier material for blueberry bioactive components: Incorporation in white chocolate. Sustainability.

[59] Rektorisova M., Tomaniova M., Hajslova J. (2022). Nut and seed butters: Lipid component quality and its changes during storage. Eur. Food Res. Technol..

[60] Aryana K., Resurreccion A., Chinnan M., Beuchat L. (2003). Functionality of palm oil as a stabilizer in peanut butter. J. Food Sci..

[61] Nadeem M., Imran M., Iqbal Z., Abbas N., Mahmud A. (2017). Enhancement of the oxidative stability of butter oil by blending with mango (*Mangifera indica* L.) kernel oil in ambient and accelerated oxidation. J. Food Process. Preserv..

